# Tuning the optimal diffusion-weighted MRI parameters on a 0.35-T MR-Linac for clinical implementation: A phantom study

**DOI:** 10.3389/fonc.2022.867792

**Published:** 2022-11-29

**Authors:** Matteo Nardini, Amedeo Capotosti, Lorenzo Nicola Mazzoni, Davide Cusumano, Luca Boldrini, Giuditta Chiloiro, Angela Romano, Vincenzo Valentini, Luca Indovina, Lorenzo Placidi

**Affiliations:** ^1^ Fondazione Policlinico Universitario “Agostino Gemelli” Istituto di Ricovero e Cura a Carattere Scientifico (IRCCS), Rome, Italy; ^2^ Azienda Unità Sanitaria Locale (AUSL) Toscana Centro, Medical Physics Unit, Prato-Pistoia, Italy; ^3^ Mater Olbia Hospital, UOS Fisica Medica, Olbia, Italy

**Keywords:** MRI, DWI, MR-linac, diffusion, ADC, MRgRT

## Abstract

**Purpose:**

This study aims to assess the quality of a new diffusion-weighted imaging (DWI) sequence implemented on an MR-Linac MRIdian system, evaluating and optimizing the acquisition parameters to explore the possibility of clinically implementing a DWI acquisition protocol in a 0.35-T MR-Linac.

**Materials and methods:**

All the performed analyses have been carried out on two types of phantoms: a homogeneous 24-cm diameter polymethylmethacrylate (PMMA) sphere (SP) and a homemade phantom (HMP) constating in a PMMA cylinder filled with distilled water with empty sockets into which five cylindrical vials filled with five different concentrations of methylcellulose water solutions have been inserted. SP was used to evaluate the dependence of diffusion gradient inhomogeneity artifacts on gantry position. Four diffusion sequences with *b*-values of 500 s/mm^2^ and 3 averages have been acquired: three with diffusion gradients in the three main directions (phase direction, read direction, slice direction) and one with the diffusion gradients switched off. The dependence of diffusion image uniformity and SNR on the number of averages in the MR sequences was also investigated to determine the optimal number of averages. Finally, the ADC values of HMP have been computed and then compared between images acquired in the scanners at 0.35 and 1.5 T.

**Results:**

In order to acquire high-quality artifact-free DWI images, the “slice” gradient direction has been identified to be the optimal one and 0° to be the best gradient angle. Both the SNR ratio and the uniformity increase with the number of averages. A threshold value of 80 for SNR and 85% for uniformity was adopted to choose the best number of averages. By making a compromise between time and quality and limiting the number of *b*-values, it is possible to reduce the acquisition time to 78 s. The Passing–Bablok test showed that the two methods, with 0.35 and 1.5 T scanners, led to similar results.

**Conclusion:**

The quality of the DWI has been accurately evaluated in relation to different sequence parameters, and optimal parameters have been identified to select a clinical protocol for the acquisition of ADC maps sustainable in the workflow of a hybrid radiotherapy system with a 0.35-T MRI scanner.

## Introduction

Magnetic resonance (MR) diffusion-weighted imaging (DWI) is a very versatile technique widely used for the diagnosis of many types of malignancy (1–5). DWI signal is sensitive to the Brownian incoherent motion of water molecules due to thermal kinetic energy and to multiple-scale microscopic physiological motions, by applying diffusion-sensitizing gradients (6). It provides a quantitative measurement of the diffusivity of water molecules by means of the apparent diffusion coefficient (ADC). Moreover, DWI is also a very valued technique for assessing the response to chemo and radiotherapy of many different types of tumor because of its sensitivity to early detection of response to therapy, even in conjunction with other MR-based imaging biomarkers (7–9). Furthermore, DWI is also used in radiotherapy for the prediction of toxicity in healthy tissues and for the construction of normal tissue complication probability models (10). Also, radiomics analyses showed promising results when applied to DW images: the extracted features have been used to train predictive models in many recent studies (11–13). In the era of magnetic resonance-guided radiotherapy (MRgRT), DWI is a perfect candidate to be included in an adaptive radiotherapy protocol (14), providing quantitative information to better adapt the daily dose distribution, considering not only the anatomical variation but also the quantitative ADC variation of the target’s tissue. Such an upgrade would greatly increase the value of the treatment in terms of personalization of the therapy. Nevertheless, DWI is still not implemented to clinically support MRgRT: in fact, up to date, few studies have been carried out on low-field MR systems to assess the reliability of DWI sequences (15–17). It is known that there are many sources of biases that influence the precision of DW images and, consequently, the reliability of the ADC estimation even in high-field MR systems devoted to medical imaging. Many of these depend on the MR system and on the acquisition sequence. The main ones are as follows:

• the signal-to-noise ratio (SNR), which decreases as the *b*-value increases (i.e., when the intensity of the diffusion gradients increases, producing a loss of phase coherence of the spins in the transverse plane and therefore a loss of SNR) (18);
• the image distortions, which strongly depend on the echo-planar readout of the most common DWI sequences and which are strongly affected by local non-uniformities of the static field (19);
• the gradient fields linearity along the three orthogonal spatial directions, which generates different effective *b*-values and image distortions (20).

Many optimization and correction strategies, as well as QA protocols, have been defined to monitor these effects and control the uncertainty of ADC measurements and possible related biases on high-field clinical MR systems (21–25). The same should be done for MR-Linacs, taking into account the peculiarities of these hybrid systems. In fact, the MR-Linac system is extremely complex: the integration of a linear accelerator and a magnetic resonance scanner in a single Faraday cage leads to several difficulties in obtaining good-quality images (26). The Linac is arranged on a circular crown arranged between two superconducting magnets that generate the field (27). Particular attention must be paid to the static field uniformity during the acquisition of images. In fact, field uniformity can be significantly affected by the movement of the ferromagnetic structure of the Linac, and image quality can be therefore dependent on the position of the Linac gantry head. For these reasons, it is necessary to characterize the MR-Linac system and optimize the DWI acquisition sequence considering the construction characteristics of the hybrid systems under examination, to obtain the desired results in terms of image quality (22). This work must be carried out by means of phantom measurements before translating the results onto the patient and also to separate the sources of uncertainty that depend on the patient (movement, breath, physiological microscopic motions, etc.) from those that depend on the MR system and on the acquisition sequence. Given this background, this study aims to assess the quality of a new DWI sequence implemented on an MR-Linac MRIdian system, evaluating and optimizing the acquisition parameters to explore the possibility of clinically implementing a DWI acquisition protocol in a 0.35-T MR-Linac.

## Materials and methods

### Sequences

All measurements were conducted in a 0.35-T MR-Linac system (MRIdian, ViewRay Inc., Mountain View, CA, USA). Since the DWI sequence is still not available clinically, all the measurements were performed in the MRI mode, disconnecting the MR scanner from the Linac and using the onboard scanner software (Syngo MR B19 DHHS, Siemens). In this modality, the MR software allowed the acquisition of DWI sequences with different types of fields of view (FOV), square or rectangular, and a slice thickness ranging from 6 to 10 mm. All the sequences used a twice-refocused spin echo (TRSE) diffusion scheme (28) with a ratio between the repetition time (TR) and echo time (TE) of 2000/5.4 and a bandwidth of 298 Hz/px. The possible choices of *b*-values for such sequences ranged continuously from 0 to 900 s/mm^2^. Moreover, an acquisition matrix of 128 × 109 pixels (pixel dimension is 2.734 × 2.734 mm^2^) was used. All images were acquired using anterior and posterior surface torso coil, considering the phantoms described in the following section.

### Phantoms

All the analyses in this work were carried out on two types of phantoms. The first was a homogeneous 24-cm diameter polymethylmethacrylate (PMMA) sphere containing a 2-mM aqueous solution of nickel chloride hexahydrate salt (NiCl_2_*6H_2_O) (Siemens Healthcare GmbH, Germany). The second was a homemade phantom (HMP) and consisted of a PMMA cylinder (183 mm diameter and 150 mm height) filled with distilled water with empty sockets into which five cylindrical vials (23 mm diameter and 100 mm height) filled with different concentrations (30, 20, 10, 5, and 1 w/w %.) of methylcellulose water solutions have been inserted.

### Diffusion gradient homogeneity

A preliminary analysis was performed to study the dependence of diffusion gradient inhomogeneity artifacts on gantry position in order to determine the best gantry angle (BGA) for DWI. For this particular analysis, SP was used and four diffusion sequences with *b*-values of 500 s/mm^2^ and 3 averages were acquired: three with diffusion gradients in the three main directions (phase direction, read direction, slice direction) and one with the diffusion gradients switched off. Measurements were repeated at four different gantry head angles of 0°, 90°, 180°, and 270°. All images were exported in DICOM format and analyzed with ImageJ software (29) (ver. 1.53f51). Image quality was evaluated by measuring the following:

• uniformity (*U*) calculated as:

*U* (%) = 1 − ((*P*_max_ − *P*_min_)/(*P*_max_ + *P*_min_)) * 100

• where *P*_max_ and *P*_min_ are the values of the maximum and minimum of the diffusion signal within the SP, the largest radius of the sphere concentric to the SP that did not include artifacts (*r*_MAX_). The latter was determined by performing visual analysis.

### Analysis of the number of averages

The dependence of diffusion image uniformity and SNR on the number of averages in the MR sequences was investigated in order to determine the best average number (BAN). This analysis was repeated for the images obtained according to four different *b*-values, i.e., 0, 300, 500, and 800 s/mm^2^, and for sequences with 1, 5, 10, and 15 averages. All measurements were carried out with the gantry head positioned at 0°. The uniformity was calculated as described before, while SNR was calculated according to AAPM guidelines (30) as follows:


$$SNR=\sqrt{2}\;S/N$$

where *S* is the mean value of the signal and *N* is the standard deviation of the background.

Secondly, an analysis of the geometric distortion as a function of the number of averages was carried out by appropriately measuring the outer diameter of the HMP in the anterior–posterior (AP) and right–left (RL) directions using images acquired with the *b*-value 500 s/mm^2^. Values have been compared to the real dimension of the phantom in order to evaluate the geometric distortion. In addition, an analysis of the dependence of the calculated ADC values on the number of averages used was carried out. Diffusion sequences were acquired on the HMP with four *b*-values (0, 300, 500, and 800 s/mm^2^), and the corresponding ADC maps were calculated using the single exponential fit of the *MRIAnalysisPak* plugin available on ImageJ software (29, 31). The distributions of the ADC values for the different methylcellulose concentrations were plotted using OriginPro “Version 2018b” (OriginLab Corporation, Northampton, MA, USA) and compared according to the number of averages in terms of mean value and standard deviation. Once the optimal parameters for the realization of a sequence applicable in clinical practice were established, diffusion images were acquired and the relative ADC maps of the HMP were calculated. These values were compared with those obtained by scanning the same HMP in a 1.5-T tomograph GE Signa HDxt (GE Healthcare, Waukesha, WI, USA) using a standard clinical sequence for diffusion imaging and the same *b*-values. The comparison was evaluated through statistical analysis using Passing–Bablok regression.

### Other analysis

The dependence of uniformity and SNR as a function of slice thickness was investigated acquiring five different diffusion sequences (BGA, BAN, 500 s/mm^2^), and slice thickness was set to 6 (minimum value allowed in the MRI protocol system for such a particular sequence), 7, 8, 9, and 10 mm, respectively.

## Results

### Diffusion gradient homogeneity

**Figure 1:**
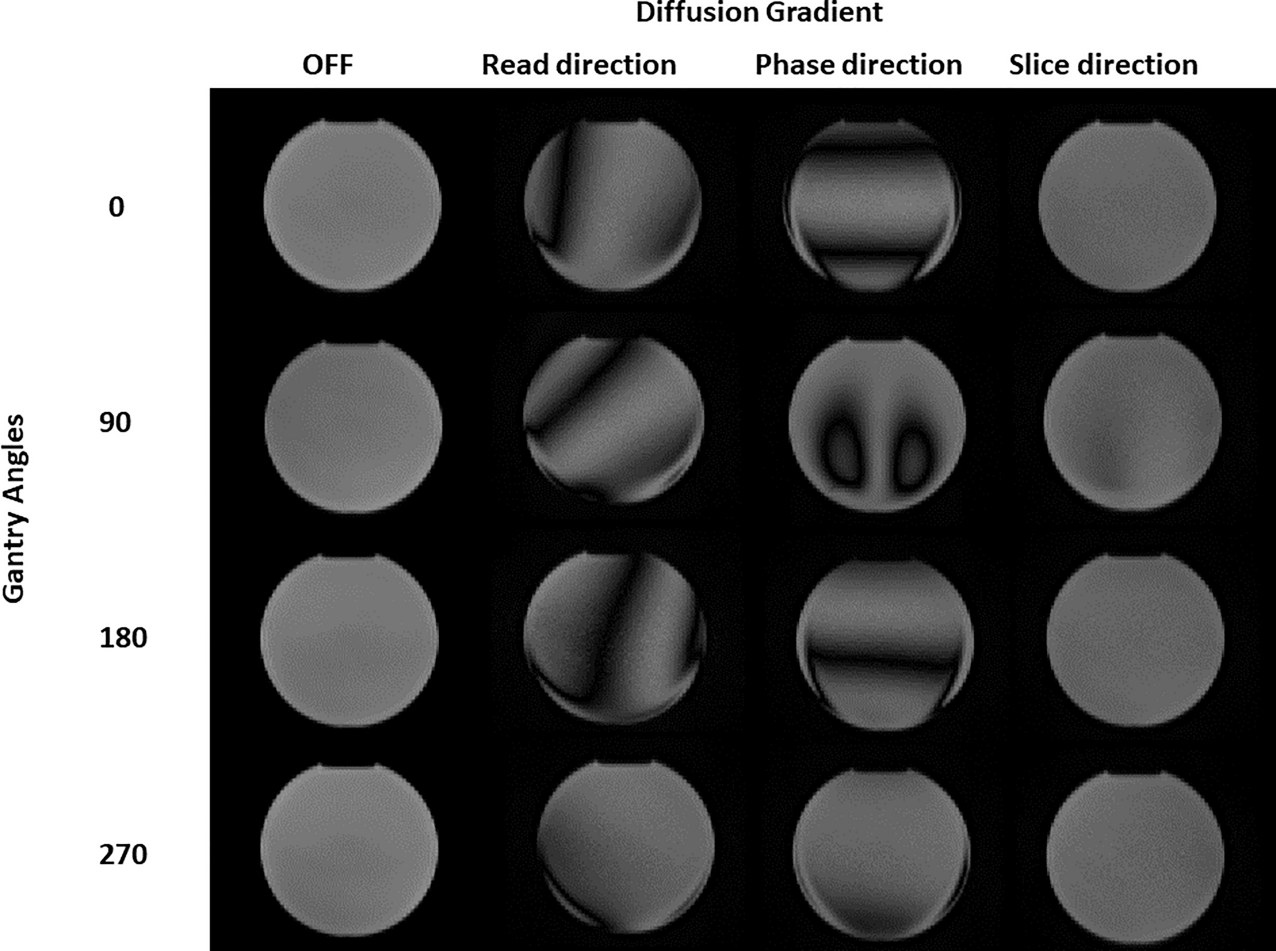
Diffusion images of the spherical phantom obtained for different gantry head positions (0, 90, 180, 270 degrees) and with different diffusion gradients (turned OFF, b-value 500 s/mm^2^ "read", "phase" and "slice" direction).

**Figure 1** shows the DWI acquisitions at different gantry angles. Both for the images acquired with the gradients turned off and the gradients turned on in the “slice” direction, there is an almost total absence of artifacts except for the 90° angle that presents a barely perceptible artifact in the center of the sphere. With regard to the images acquired with the gradients turned on in the “read” and “phase” directions, the copious presence of inhomogeneous gradient artifacts can be noted in all gantry angles. These visual considerations are reinforced by the data in **Table 1** which shows the results of the computed uniformity (*U*) and the *r*_MAX_ value at different gantry angles for the SP DWI. In this table, we can see that the uniformity reaches its maximum values (93.2, 84.8, 91.4, and 88.0 with gantry angle at 0°, 90°, 180°, and 270°, respectively) with the gradients off and with the gradients on in the “slice” direction (90.3, 76.3, 90.0, and 83.8 with gantry angle at 0°, 90°, 180°, and 270°, respectively). For the images acquired with the gradients in the “read” and “phase” directions, the uniformity has almost always the lowest values. Concerning the *r*_MAX_ value, expressed in millimeters, **Table 1** reports its value related to the images with the gradients off and in those with the gradients turned on in the “slice” direction; the maximum value is reached at 120 mm (the SP has in fact a diameter of 240 mm). On the other hand, for images acquired with the gradients in the “read” and “phase” directions, *r*_MAX_ minimum values are between 0 and 73 mm. The worst situation was observed with the gantry angle at 180° where *r*_MAX_ for the “read” and “phase” gradients are zero because of the evident artifacts that cross the image right in the middle of the FOV. The overall result of this analysis identifies “slice” as the optimal gradient direction and 0° as the best gradient angle, to acquire high-quality artifact-free DWI images.

**Table 1:** Values of uniformity (U) and maximum radius of artifact-free ROI for the images in **Figure 1**.

Gantry angle	Gradient	*U* (%)	*r*_MAX_ (mm)
0	OFF	93.2	120
	b500 read	0.0	48
	b500 phase	0.0	42
	b500 slice	90.3	120
90	OFF	84.8	120
	b500 read	0.0	31
	b500 phase	0.0	20
	b500 slice	76.3	31
180	OFF	91.4	120
	b500 read	0.0	0
	b500 phase	0.0	0
	b500 slice	90.0	120
270	OFF	88.0	120
	b500 read	0.0	83
	b500 phase	39.4	73
	b500 slice	83.8	120

Values are calculated and measured for different gantry angles and different diffusion gradient directions (gradients OFF, b-value of 500 s/mm^2^ “read,” phase,” and “slice” directions, 3 averages).

### Analysis of the number of averages

**Figure 2:**
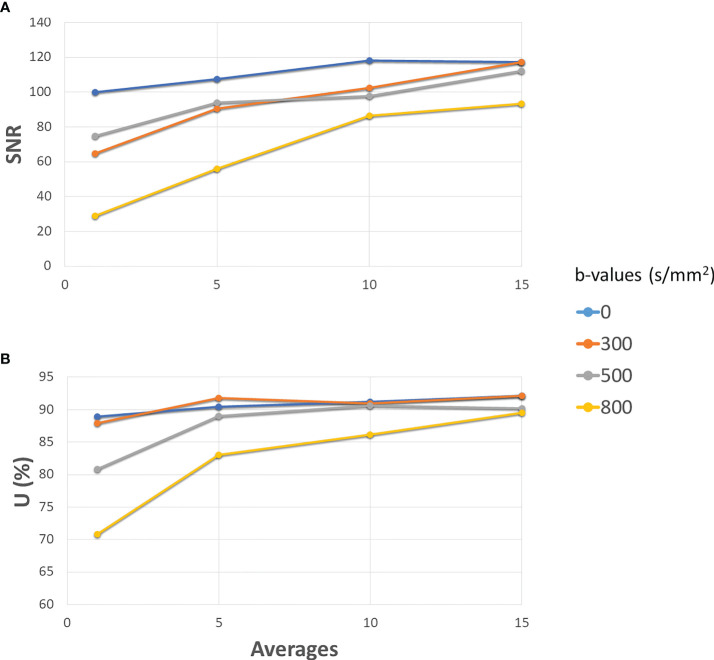
Development of SNR **(A)** and Uniformity **(B)** as the number of averages increases for different b-values. The solid blue line is for 0 s/mm^2^ (gradients off), orange for 300 s/mm^2^, grey for 500 s/mm^2^ and yellow for 800 s/mm^2^. All the gradients have been set to slice direction.

**Figure 2** depicts the SNR (panel **A**) and the uniformity (panel **B**) as a function of the number of averages when varying the *b*-values. Both the SNR ratio and the uniformity increase with the number of averages. For the *b*-value 0 s/mm^2^, SNR has the minimum value of 100 (1 average) and assumes the maximum value (118) at 10 averages. For the *b*-value 300 s/mm^2^, SNR varies continuously from 64 (1 average) to 117 (15 averages). Similarly, for the *b*-value 500 s/mm^2^, SNR varies continuously from 74 (1 average) to 112 (15 averages). The *b*-value 800 s/mm^2^ shows obviously lower values than the others, ranging from a minimum of 29 (1 average) to a maximum of 93 for 15 averages, reaching a value of 86 for 10 averages. For the lowest *b*-values (0 and 300 s/mm^2^), we find a high value of uniformity and SNR (as observed with 15 averages) even using few averages (1 or 5), while for the highest ones (500 and 800 s/mm^2^), optimal values are reached starting from 10 averages. On the basis of these considerations, a threshold value of 80 for SNR and 85% for uniformity was adopted to choose the best BAN, which is dependent on the *b*-value: a smaller number of averages (3) can be used for the lowest *b*-values and a larger number of averages (10) must be used for the highest *b*-values. **Table 2** reports the times taken by the sequences for the acquisition of a single slice and for a stack of 6 slices, according to the number of averages. The time reported is relative to the acquisition of an image with only one *b*-value; to obtain the total duration of a sequence used to generate an ADC map, the times necessary to obtain all the single *b*-values involved must be added together.

**Table 2:** Time required to collect images with 1 (first column) or 6 (second column) slices varying the number of averages of the sequence.

Averages	Time/1 slice (s)	Time/6 slices (s)
1	4	10
5	12	31
10	22	57
15	32	83

**Figure 3:**
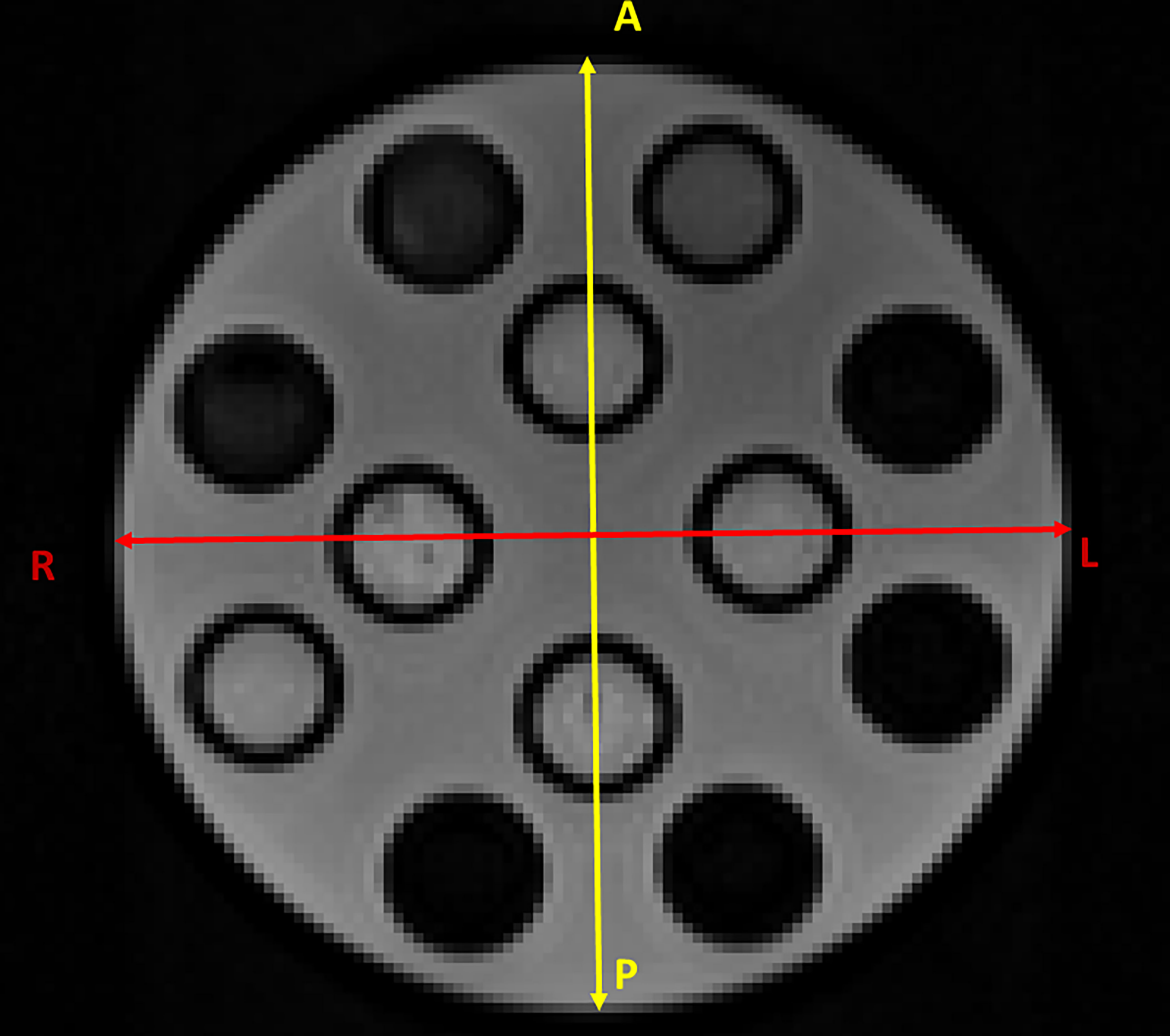
Section of the HMP used to calculate the two diameters in the AP (in yellow) and RL (in red) directions.

**Figure 3** shows the section of the HMP used to calculate the two diameters in the AP (in yellow) and RL (in red) directions. **Table 3** shows the results of the measurements and the deviations from the expected value (Δ) as a function of the number of averages. It can be seen that the distance from the expected value is always below 1 mm except for the values for images with only one mean. In this case, in fact, there is a difference of 2.9 mm for the AP direction and 1.36 mm for the RL direction.

**Table 3:** Value of the diameter measurements in the two directions (AP second column and RL third column) expressed in millimetres.

Averages	AP diameter (mm)	RL diameter (mm)	ΔAP (mm)	ΔRL (mm)
1	180.1	181.64	2.90	1.36
5	183.21	182.94	−0.21	0.06
10	183.25	182.82	−0.25	0.18
15	183.77	183.51	−0.77	−0.51

In the fourth and fifth column the difference with the expected value. All images are acquired using TRSE sequences with TR/TE = 2000/5.4 and a b-value of 500 s/mm^2^ in slice direction.

In the fourth and fifth columns, differences with the expected values are shown. All images are acquired using the TRSE sequences with TR/TE = 2000/5.4 and a *b*-value of 500 s/mm^2^ in the slice direction.

**Figure 4:**
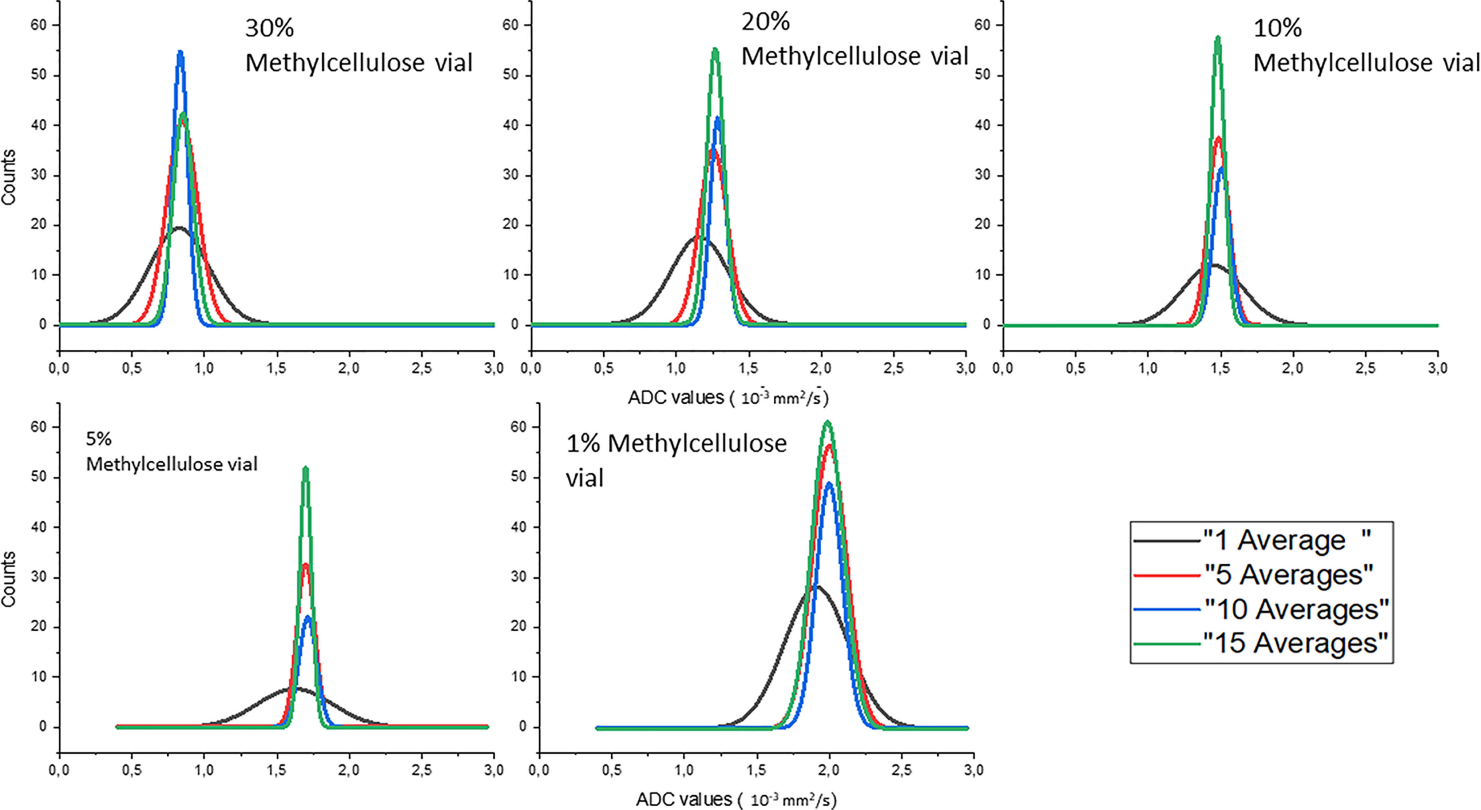
Gaussian fits of the distributions of the ADC values of the different concentrations of methylcellulose in the HMP obtained for different numbers of averages.

Gaussian fits of the distributions of the ADC values of the different concentrations of methylcellulose in the HMP obtained for different numbers of averages are shown in **Figure 4**. The ADC values are given in 10^−3^ mm^2^/s, and we can see in solid black line the values obtained for 1 average, in red those for 5 averages, in blue those for 10 averages, and in green those for 15 averages.

In **Table 4**, we can see the parameters of the Gaussian fits for ADC value distribution. Mean value (*x*_m_) and standard deviation (*σ*) are reported in 10^−3^ mm^2^/s for all methylcellulose concentrations and for all numbers of averages considered in the analysis. It can be seen that the value of the ADCs remains constant except for the values obtained for 1 average which are significantly lower. On the other hand, the standard deviation decreases its value as the number of averages increases except for the 1% concentration which shows similar but slightly higher values from 5 to 10 averages.

**Table 4:** Parameters of Gaussian fits for ADC value distributions for all methylcellulose concentrations and all averages analyzed.

Methylcellulose %		1 average	5 averages	10 averages	15 averages
30	*x*_m_	0.88 ± 0.02	0.90 ± 0.01	0.89 ± 0.01	0.91 ± 0.01
	*σ*	0.41 ± 0.03	0.22 ± 0.01	0.10 ± 0.01	0.10 ± 0.01
20	*x*_m_	1.22 ± 0.02	1.31 ± 0.01	1.32 ± 0.01	1.33 ± 0.01
	*σ*	0.40 ± 0.02	0.19 ± 0.01	0.11 ± 0.01	0.11 ± 0.01
10	*x*_m_	1.48 ± 0.02	1.52 ± 0.01	1.52 ± 0.01	1.51 ± 0.01
	*σ*	0.42 ± 0.03	0.14 ± 0.01	0.12 ± 0.01	0.10 ± 0.01
5	*x*_m_	1.66 ± 0.02	1.73 ± 0.01	1.74 ± 0.01	1.73 ± 0.01
	*σ*	0.50 ± 0.03	0.12 ± 0.01	0.12 ± 0.01	0.10 ± 0.01
1	*x*_m_	1.91 ± 0.02	1.99 ± 0.01	1.99 ± 0.01	1.99 ± 0.01
	*σ*	0.44 ± 0.03	0.18 ± 0.01	0.19 ± 0.01	0.15 ± 0.01

Mean values and standard deviations are reported in 10^−3^ mm^2^/s.

**Table 5** reports the SNR and uniformity values as a function of the slice thickness: both values do not vary significantly. The increase in slice thickness results in an increase in SNR (from 93.8 to 96.1) and uniformity (from 89% to 92%).

**Table 5:** SNR and uniformity as a function of the slice thickness.

Slice thickness (mm)	SNR	*U* (%)
6	93.8	89
7	94.1	89
8	94.7	90
9	95.4	91
10	96.1	92

Values are calculated on the images of the SP acquired with a sequence at BGA, with gradient in the “slice” direction and a b-value of 500 s/mm^2^.

**Figure 5:**
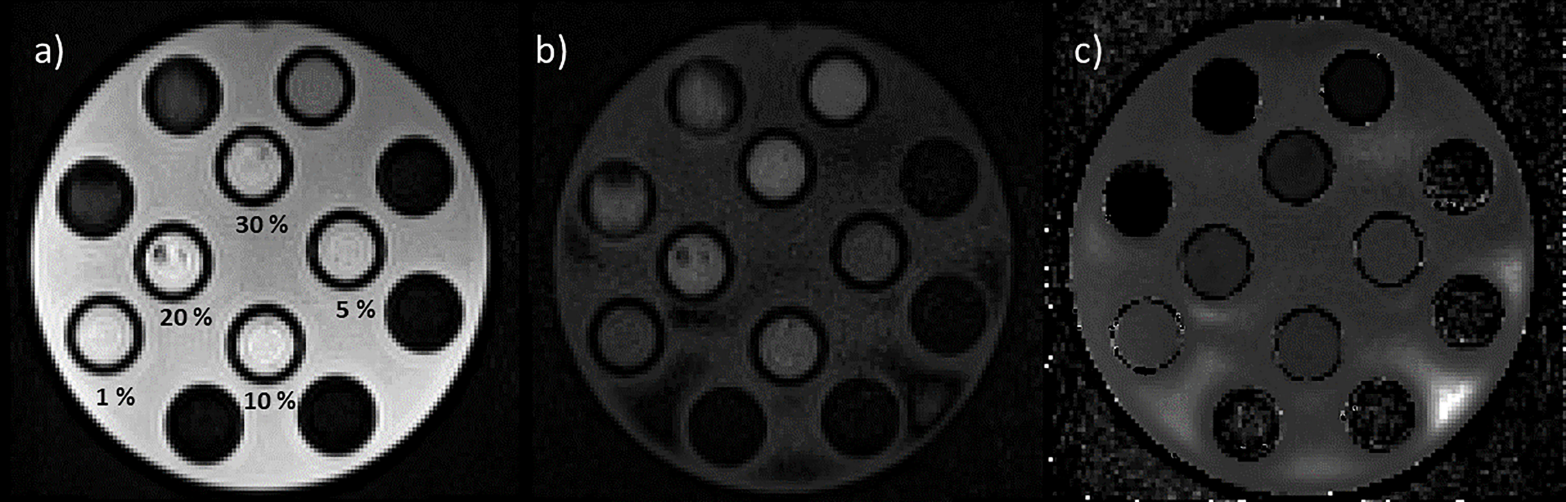
Images of the HMP acquired using diffusion sequences with b-values set to 0 s/mm^2^, 3 averages (a) and 800 s/mm2, 10 averages (b). In panel “c” is reported the calculated ADC map. In panel “a” are also reported the values of the concentrations of methylcellulose solution below the relative vial.

As shown in **Figure 5**, DWI was then acquired on HMP, and the corresponding ADC map was calculated using two *b*-values (0 and 800 s/mm^2^), with the number of averages set to 3 and 10, respectively, while slice thickness was maintained to 6 mm. These settings allow to acquire a single slice in 26 s and a stack of 6 slices, which would give a volume of 350 × 294 × 36 mm^3^, in 78 s (3 averages in 21 s for *b*-value at 0 s/mm^2^ and 10 averages in 57 s for *b*-value at 800 s/mm^2^). All sequences were acquired at the BGA.

**Figure 6:**
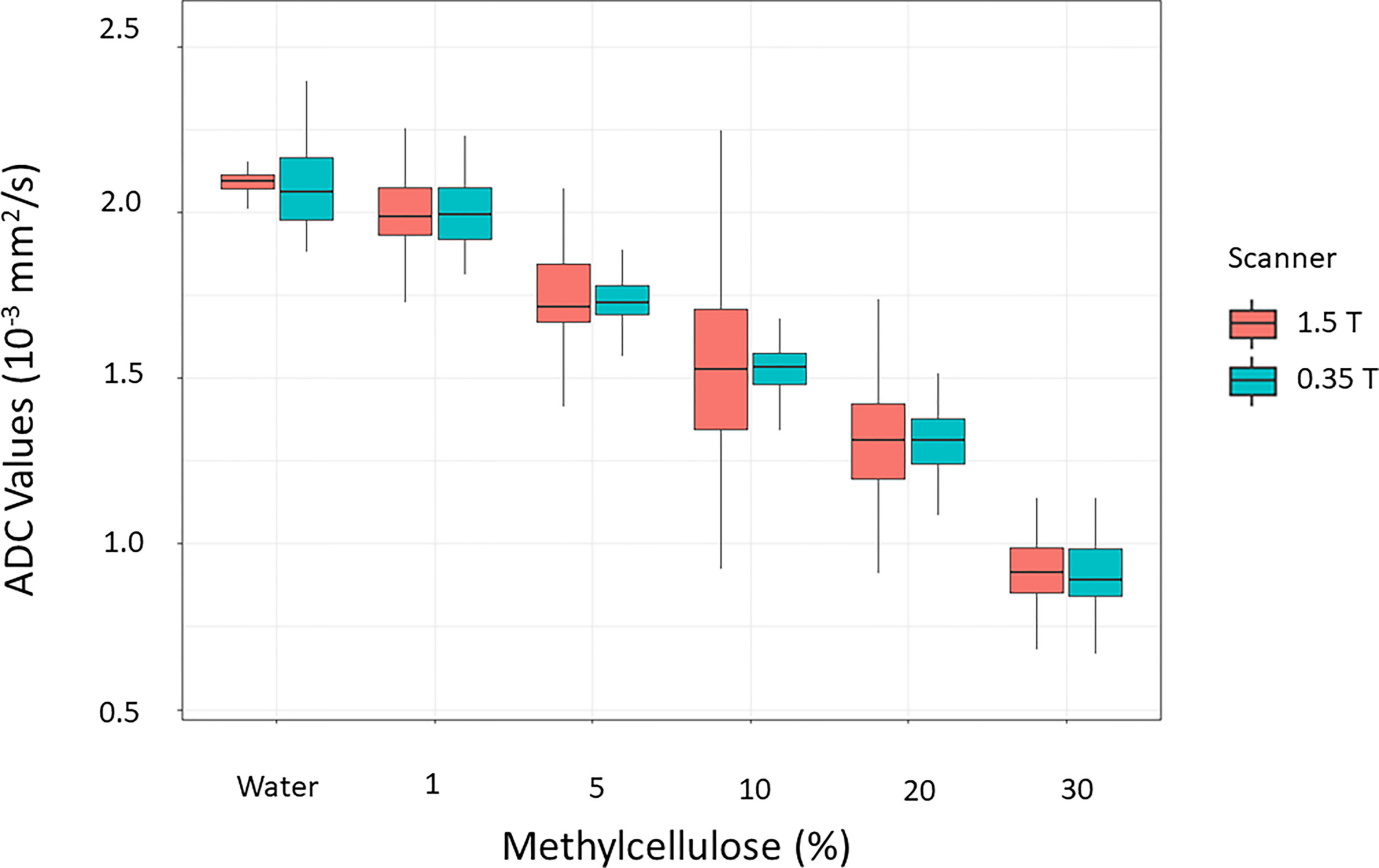
Distributions of the values of the ADC coefficients calculated from the images acquired on the scanner at 0.35 T (cyan box) in comparison with those acquired with a homologous sequence on a diagnostic scanner at 1.5 T (orange box) for the various concentrations of the methylcellulose solutions present in the HMP.

**Figure 6** reports the boxplots of the ADC coefficient values computed from the images acquired on the scanner at 0.35 T (cyan box) and on a diagnostic scanner at 1.5 T (orange box), for the various concentrations of the methylcellulose solutions present in the HMP.

**Figure 7:**
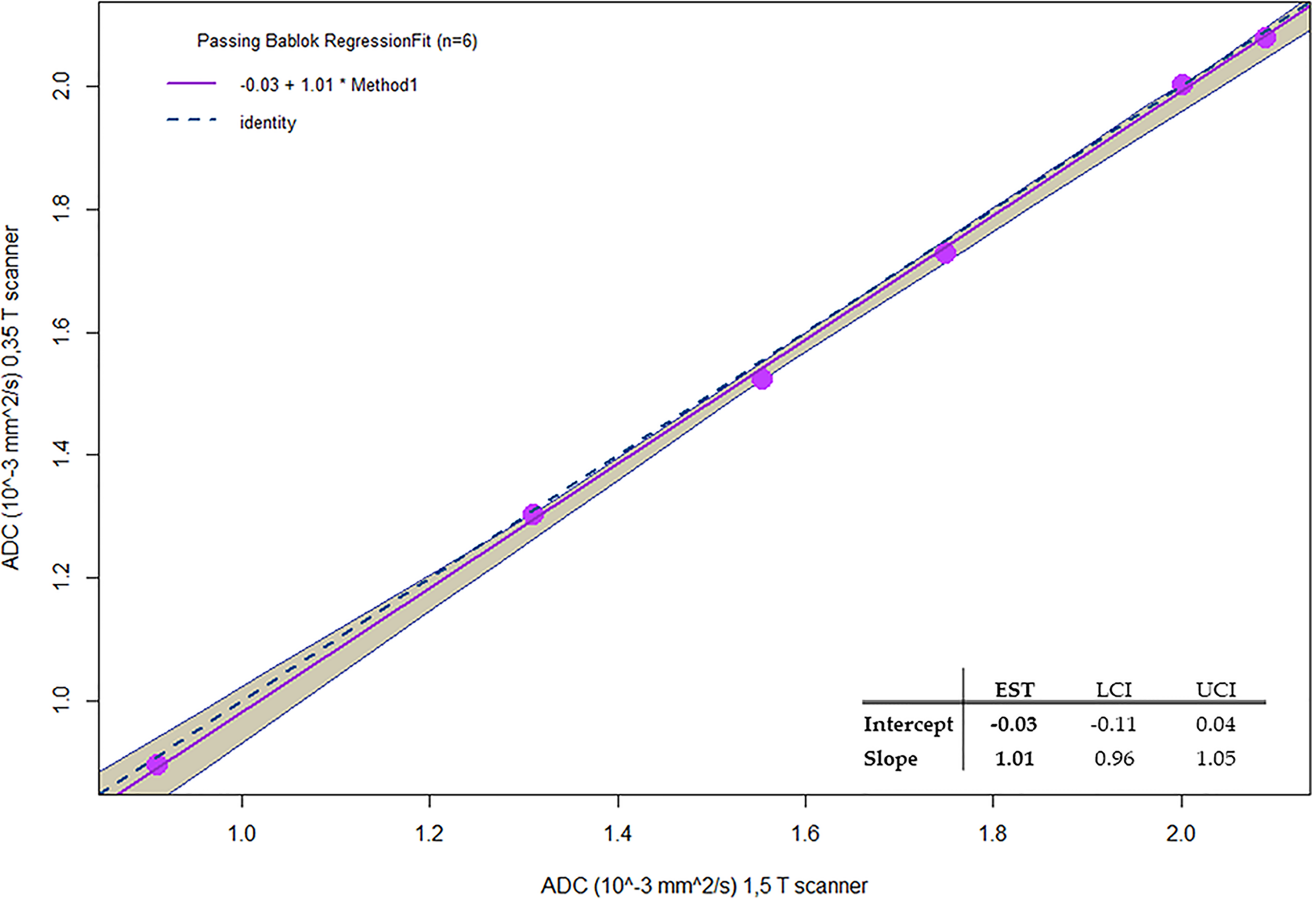
Passing-Bablok regression for comparison of ADC values of Methylcellulose concentrations for 1.5 T and 0.35 T scanners.

**Figure 7** reports the Passing–Bablok regression for comparison of ADC values of methylcellulose concentrations for 1.5 and 0.35 T scanners: the comparison showed a slope value of 1.01 (95% CI: 0.96 to 1.05) and an intercept value of −0.03 (95% CI: −0.11 to 0.04).

In **Table 6**, the ADC mean values and relative standard deviations for the various concentrations of methylcellulose and distilled water are reported. As it would be desirable, no significant differences between the ADC coefficient distributions obtained on the two different scanners have been noticed: the most significative variation is 0.03 10^−3^ mm^2^/s for 10% of methylcellulose concentration, while it shows no difference for the 1% concentration. The agreement between couples of relative ADC mean values can be appreciated by reading the *p*-values in the fifth column which are all above the significance level. In **Table 7**, we summarize the optimal acquisition parameters for our center.

**Table 6:** Mean values of ADC (10^−3^ mm^2^/s) obtained for different methylcellulose concentrations and distilled water.

% Methylcellulose	Scanner	Mean ADC Value	St.Dev.
30	1.5 T	0.91	0.10
	0.35 T	0.90	0.11
20	1.5 T	1.31	0.19
	0.35 T	1.30	0.10
10	1.5 T	1.55	0.29
	0.35 T	1.52	0.08
5	1.5 T	1.75	0.16
	0.35 T	1.73	0.07
1	1.5 T	2.00	0.13
	0.35 T	2.00	0.11
Distilled water	1.5 T	2.09	0.03
	0.35 T	2.08	0.13

Results are reported for both scanners used to acquire images: 1.5 T diagnostic scanner and 0.35 T MRIdian integrated scanner.

**Table 7:** Acquisition parameters for ADC measurements.

Topic	Parameters
Diffusion scheme	TRSE
Diffusion gradient direction	Slice
TR/TE	2000/5.4
Gantry angle	0
Number of averages	At least 3 (*b*-value-dependent)
Max *b*-values	800 s/mm^2^
Slice thickness	6 mm (lower possible)

## Discussion

In this study, the quality of DWI was evaluated in relation to different sequence parameters to identify the optimal parameters and create a clinical protocol for the acquisition of ADC maps sustainable in the workflow of a hybrid radiotherapy system with a 0.35-T MRI scanner. Initially, the dependence of the image quality on the position gantry angle was studied, observing the use of diffusion gradients in the “read” and “phase” directions which produced images with a large number of artifacts; however, they could not be used for the calculation of ADC maps. As regards the “slice” direction, good-quality images were obtained for most of the gantry angles investigated in this study (except for 90°). A similar behavior was also found in Pieniazek et al. (32) who used a 0.2-T MR scanner and only slice direction due to system limitations. In previous studies investigating the DWI acquisition on a 0.35-T MR-Linac system, there is no mention of the gradients’ direction and the gantry angle used (33, 34). Although is not clearly visible in **Figure 1**, the quantitative analysis in **Table 1** shows the differences in acquisitions with diffusion gradients in the “slice” direction for the four gantry angles (0°, 90°, 180°, and 270°) that led to the choice of 0° as the best gantry angle. Based on these findings, a clinical protocol will be designed using 0° as the best gantry angle and slice as the diffusion gradient direction. Regarding the analysis of the number of averages, it was confirmed that there was also an increase in both SNR and uniformity when the number of averages is increased: based on this, the different numbers of averages depending on the *b*-value acquired were considered. By making a compromise between time and quality and limiting the number of *b*-values used for the calculation of the ADC maps to 2, it is possible to reduce the acquisition time to 78 s. This time reduction can be considered a satisfactory result since the duration of the typical diagnostic scanner sequences ranges from 40 s to 3–4 min depending on the need (35) and patient compliance (36). Our results in terms of stability analysis of ADC values as a function of averages are in line with those reported in a previous experience recently published (37, 38). Since the results obtained for the variation of SNR and uniformity showed insignificance to minor deviations when varying slice thickness, we did not find it useful to proceed with an analysis of the stability of ADC coefficient values as a function of slice thickness. The optimal sequence designed was finally tested acquiring DWI on the HMP and calculating ADC values. Such maps were compared with those obtained with acquisitions on a 1.5-T diagnostic scanner on the same phantom, obtaining good agreement which is desirable. As can be seen in **Figure 6**, there is a certain difference in the standard deviations of the values obtained using the two different scanners: a probable explanation can be found in the inhomogeneity of the methylcellulose solutions since some vials contain more inhomogeneous solution than the others due to small lumps or small air bubbles. These are detected, when present, by the higher resolution of a 1.5-T scanner leading to a higher standard deviation. This is a limitation of this study and can be overcome by using a different polymer in the solution [like polyvinylphenol (PVP)] to make it more homogenous. As far as the authors know, this represents one of the first studies on diffusion sequences carried out on a 0.35-T system using a phantom. A comparable study was proposed by Lewis et al., who investigated the geometric distortion as a function of gantry angles (38). The substantial differences mainly involved two aspects: the first merely concerns the parameters used for the sequence. Lewis et al. used an EPI diffusion scheme and made no mention of using particular gradient directions. The other aspect concerns the phantom: Lewis et al. made use of a commercial NIST phantom for DWI, while our measurements were carried out using a homemade phantom that is easily replicable and cheap. Lewis et al. found a difference in ADC values when comparing the scanners at 0.35 T with those at 1.5 and 3 T, while in our study, there is a good agreement in ADC values calculated with images acquired in the 0.35- and 1.5-T scanners as shown by the Passing–Bablok regression analysis. A possible reason for this discordance lies in the different diffusion schemes used and probably in the choice of the different *b*-values chosen for the sequences. In addition, a similar study was published on an MR-Linac system with a static magnetic field at 1.5 T by Kooreman et al. (39). In this multicenter study (6 MR-Linac system scanners), the spatial dependence of the ADCs was evaluated using a cylindrical phantom. Similar to the present work, Kooreman et al. also found images affected by artifacts for acquisitions with diffusion gradients that were not in the z-direction (our “slice” direction). Although they found the presence of these artifacts, they did not render the images unusable but only forced them to define a confidence zone around the isocenter (7 cm radius) for the calculation of ADC values. In our case, however, if we had to calculate the same kind of confidence zone, we would have found a null surface. This study obviously has all the limitations of a single-center study. This calls for a multicenter evaluation study, involving other MR-Linac systems with a static magnetic field at 0.35 T, to characterize the gradient inhomogeneities in a machine-independent manner to understand their nature and make the necessary corrections.

In conclusion, the present study identified the optimal parameters to obtain high-quality diffusion-weighted MR images on a 0.35-T MR-Linac system.

## Data availability statement

The original contributions presented in the study are included in the article/supplementary material. Further inquiries can be directed to the corresponding author.

## Author contributions

MN provided writing (draft), data curation and formal analysis. MN, LNM provided methodology. MN, LNM, LP provided conceptualization. LB, GC, AR provided resources and writing (review). AC, LNM, DC, LP provided supervision, validation and writing (review). LP, LI, VV provided project administration and funding acquisition.

## Conflict of interest

LB, LP, and DC received research grants and personal fees from ViewRay.

The remaining authors declare that the research was conducted in the absence of any commercial or financial relationships that could be construed as a potential conflict of interest.

## Publisher’s note

All claims expressed in this article are solely those of the authors and do not necessarily represent those of their affiliated organizations, or those of the publisher, the editors and the reviewers. Any product that may be evaluated in this article, or claim that may be made by its manufacturer, is not guaranteed or endorsed by the publisher.
